# How sensitive and specific is the visual diagnosis of sarcoptic mange in free-ranging Iberian ibexes?

**DOI:** 10.1186/s13071-019-3665-7

**Published:** 2019-08-15

**Authors:** Marta Valldeperes, José Enrique Granados, Jesús María Pérez, Inmaculada Castro, Arián Ráez-Bravo, Paulino Fandos, Jorge Ramón López-Olvera, Emmanuel Serrano, Gregorio Mentaberre

**Affiliations:** 1 Wildlife Ecology & Health group (WE&H) and Servei d’Ecopatologia de Fauna Salvatge (SEFaS), Departament de Medicina i Cirurgia Animals, Universitat Autònoma de Barcelona (UAB), 08190 Bellaterra, Barcelona Spain; 2 Espacio Natural Sierra Nevada, Carretera Antigua de Sierra Nevada, Km 7, Pinos Genil, 18071 Granada, Spain; 3 Departamento de Biología Animal, Biología Vegetal y Ecología, Universidad de Jaén, Campus Las Lagunillas, s.n, 23071 Jaén, Spain; 4 Agencia de Medio Ambiente y Agua de Andalucía, 41092, Isla de la Cartuja, Sevilla, Spain; 5 Dipartimento di Scienze Veterinarie, Universitá di Torino, Grugliasco, Torino, Italy; 6 Departament de Ciència Animal, Escola Tècnica Superior d’Enginyeria Agraria (ETSEA), Universitat de Lleida (UdL), 25098 Lleida, Spain

**Keywords:** *Capra pyrenaica*, *Sarcoptes scabiei*, Selective culling, Visual diagnostic, Wildlife disease management

## Abstract

**Background:**

Sarcoptic mange is a broadly distributed parasitic disease caused by *Sarcoptes scabiei* that affects wild mammals from all over the world, including the Iberian ibex (*Capra pyrenaica*). Selective culling of the scabietic individuals is the main management measure for disease control in Iberian ibex populations. Although visual identification of mange-compatible lesions is the reference method to decide the target individual, both false negative and positive cases are common in the wild. The aim of this work is to determine the sensitivity (SE), and the specificity (SP) of selective culling after evaluating 403 ibexes hunted in the Sierra Nevada Nature Space for sarcoptic mange control between 2002 and 2015.

**Methods:**

A combination of skin scrapings and potassium hidroxide (KOH) skin digestion was used for sarcoptic mange diagnosis. Generalized linear models (GLM) were used to assess the effects of sex, age (juveniles and adults) and period of the year (wet and dry periods) on the SE and SP of the visual diagnosis method.

**Results:**

The SE obtained for the visual determination of scabietic ibexes was 87.14%, whereas the SP was 60.71%. According to our model selection, SE of the visual diagnosis was explained by the additive effects of age and the period of the year. In fact, SE was lower in juveniles (64.76%) than in adults (94.26%) and during the dry period (73.44%) as compared to the wet period (92.09%). On the other hand, SP was best explained by the GLM including the additive effects of sex and the period of the year. The visual diagnosis of sarcoptic mange resulted less specific in females (22.73%) than in males (74.19%) and during the wet (55.22%) than in the dry period (82.35%).

**Conclusions:**

Maximizing SE and SP is essential to achieving a high rate of removal of affected individuals from the environment without eliminating potentially resistant individuals. Selective culling must be conservative during the wet period and with females due to the lower SP. Conversely, visual diagnosis of scabietic juveniles and during the dry period has to be improved, due to the lower SE.

## Background

**Fig. 1:**
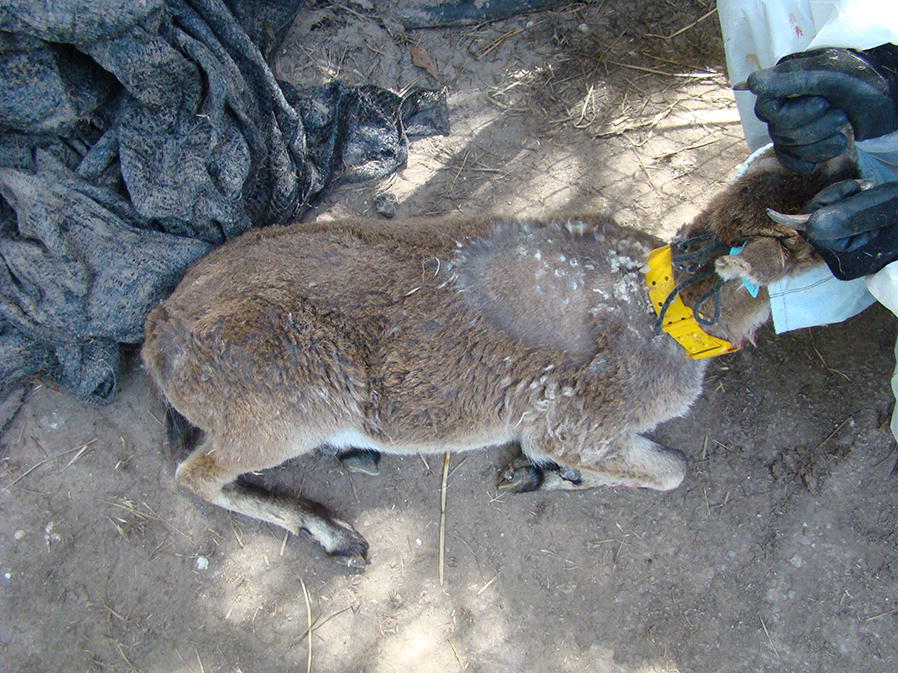
Iberian ibex recovering from sarcoptic mange experimental infestation in SNNS showing healing lesions. New hair is growing within the old lesions

Sarcoptic mange is a parasitic disease caused by *Sarcoptes scabiei* reported in at least 104 mammal species from 27 families and 10 orders, responsible for wildlife population collapses all over the world [1–3]. These include: carnivores, e.g. coyote (*Canis latrans*) [4], cheetah (*Acinonyx jubatus*) [5], Eurasian lynx (*Lynx lynx*) [3, 6], red fox (*Vulpes vulpes*) [6, 7] and pine marten (*Martes martes*) [6]; marsupials, e.g. wombat (*Vombatus ursinus*) [8, 9], koala (*Phascolarctos cinereus*) [10]; and ungulates, e.g. chamois (*Rupicapra pyrenaica*) [11] and Iberian ibex (*Capra pyrenaica*) [12, 13], among others. Despite the fact that it has led some populations almost to extinction [7, 8, 12, 13], survival of scabietic animals is not rare [14, 15] (Fig. 1). Once a population is affected by sarcoptic mange, it remains endemic re-emerging cyclically with lower virulence [3, 12, 16].

Sarcoptic mange pathology depends on the host and the environmental conditions. It is typically seasonal [17], peaking in late winter and spring, when low temperatures and high humidity favour the survival and reproduction of adult mites [18, 19]. Sexual differences in the prevalence of sarcoptic mange [17, 20] could be explained by gender differences in the immune response against the mite [21, 22]. In addition, prevalence differences amongst age classes have also been reported in mammals, including wild ungulates, with adults often being more susceptible [11, 12, 16]. As a result, sarcoptic mange outbreaks commonly happen in early spring, mostly affecting adult males [17, 20].

Measures for sarcoptic mange control are often implemented to mitigate the impact of the disease on wildlife populations, as well as for social and political concerns. In field conditions, these measures are generally based on the selective culling of individuals with sarcoptic mange compatible lesions and pruritus, as the main clinical sign [3, 12, 17, 23, 24], as well as the administration of antiparasitic drugs such as ivermectin or amitraz [5, 6, 10, 12]. The characteristic lesions of sarcoptic mange include erythematous eruptions, papules, seborrhoea, severe alopecia, crusts, hyperkeratosis and skin lichenification, dermal fissures, eyelid and lip inflammation and, finally, systemic signs as dehydration and emaciation [1, 2, 12, 13, 15].

It is presumed that individuals showing a high proportion of damaged skin commonly carry higher mite burden than their less affected counterparts. However, the individual hypersensitivity response to a given parasite burden is in great measure what determines the outcome of sarcoptic mange infestation [2]. On the other hand, mite burden for a given skin surface can vary seasonally [25].

**Fig. 2:**
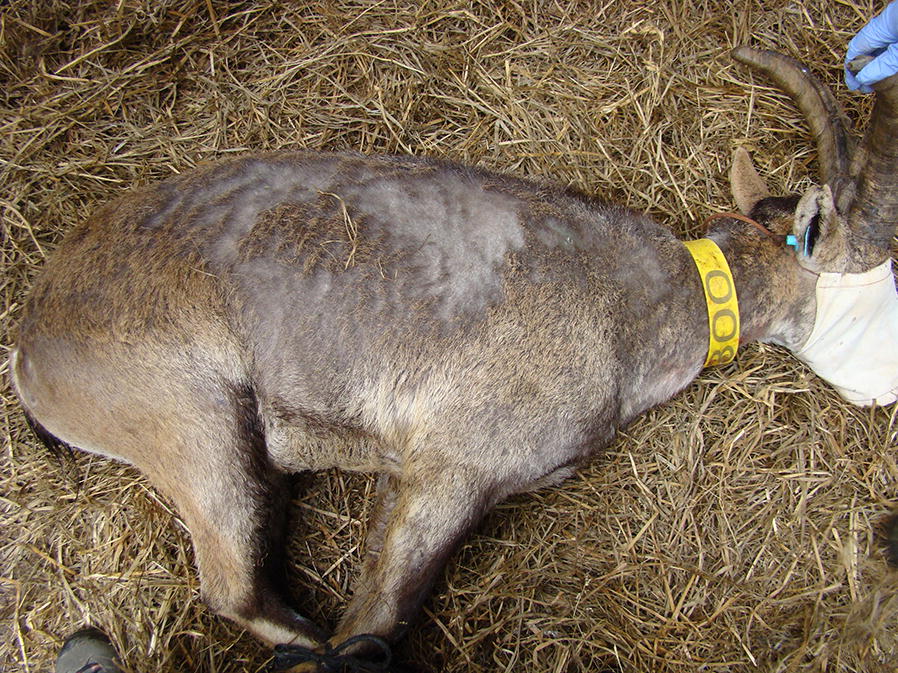
Iberian ibex showing severe alopecia; however, skin scrapings and skin digestions were negative for *S. scabiei* detection

Nevertheless, other skin disorders also result in alopecia, crusts and scales, making it difficult to confirm the visual diagnosis of sarcoptic mange in severely affected individuals [26] (Fig. 2). These alterations may include pemphigus complex, dermatophytosis and dermatophilosis, other fungal and bacterial dermatitis, nutritional imbalances, chorioptic mange, louse, flea and tick infestations [27, 28], and photo-sensitization, among others [29]. Furthermore, in early summer ibexes molt fur to get the summer coat, presenting an unhealthy appearance in the eyes of an unexperienced observer. This emphasizes the need to confirm the visual diagnosis with additional tests, such as potassium hydroxide (KOH) digestion of the skin with observation of the mites, ova or faecal pellets under microscopic scrutiny [30, 31].

Visual diagnosis of sarcoptic mange is the reference field method used not only in wildlife disease research [32] but also in actions for disease control in free ranging populations confirmed outbreaks [33]. Few efforts, however, have been made to determine sensitivity (SE) and specificity (SP) of this “visual test and slaughter” method. Although there is a need to assess the accuracy of this procedure [25, 31], the effects of age, sex and season on the SE and SP of visual diagnosis of sarcoptic mange on the field remain untested.

The Iberian ibex is a medium-sized mountain ungulate and an excellent model to evaluate the SP and SE of field visual diagnosis of sarcoptic mange due to: (i) high sexual dimorphism; (ii) easy determination of age; and (iii) existence of different stages of mange infestation in wild populations. Since the first detection of sarcoptic mange in 1992 [17, 27] in the Iberian ibex population of the Sierra Nevada Natural Space (Including both the National and Natural Park, SNNS hereafter), selective culling of individuals showing sarcoptic mange compatible lesions has been applied, together with the habitual hunting plan for population management purposes. On the other hand, individuals in this population have a strong seasonal pattern of body reserves [34] that is partly driven by mange infestation [20], particularly during winter [35].

The aim of this study is to determine the SE and SP of the visual diagnosis of sarcoptic mange in Iberian ibex spotted during field prospections in the SNNS, and to explore the individual (age and sex) and environmental (period of the year) effects on the SE and SP of this method. The information obtained should be useful to improve strategies oriented to improve sarcoptic mange management and control strategies in wild ungulate populations.

## Methods

This work was performed with data obtained for the study of Pérez et al. [25] and with data collected from the management programme of the Iberian ibex in SNNS.

### Animals and sampling

Four hundred and three Iberian ibexes culled between 2002 and 2015 in the SNNS, southern Spain (36°00′–37°10′N, 2°34′–3°40′W), were included in this work. These were ibexes presenting sarcoptic mange compatible lesions, but also apparently healthy ones that were killed either as part of the usual population management programme or through game hunting (females, kids, juveniles and trophy-hunted males).

The ibexes were first visually classified in five categories of sarcoptic mange affectation (0, no skin lesions; 1, 1–25% of the skin affected; 2, 25–50%; 3, 50–75%; 4, > 75%) and then culled as part of previously mentioned population management programme in the SNNS. In addition, sex, age (kid, 0 years; yearling, 1 to 2 years; juveniles, 2 to 4 years; and adults, > 4 years), season and localization were recorded for every ibex. Finally, the diagnosis of sarcoptic mange was confirmed by skin scrapings and/or KOH digestion (when negative scraping was obtained). The combination of these two methods was considered as the gold standard in this study. Detailed information about the diagnostic procedures can be found in Pérez et al. [25]. Fifty-six ibexes showing 1–25% of affectation (category 1) were excluded from the study because of the potential bias during the visual diagnosis, as they are the hardest to classify and could thus lead to a higher error in field conditions [13]. Thus, only 347 Iberian ibexes were finally included in the study (3 kid females and 5 kid males, 13 yearling females and 19 yearling males, 22 juvenile females and 116 juvenile males, 78 adult females and 70 adult males, 29 with unrecorded sex and/or age).

### Statistical procedures

To assess the sex, age and period of the year effects on the SE and SP of our visual diagnosis method for detecting scabietic ibexes we used specific generalized linear models (GLM) with a binomial error distribution and “logit” link function. The GLM for SE used a binomial variable as a response variable: 1 for false positive individual and 0 otherwise; whereas the GLM for SP used: 1 for false negative individual and 0 otherwise. A false positive (FP) was considered the case where an Iberian ibex was visually diagnosed as scabietic, but then was both negative to skin scrapings and skin digestions. A false negative (FN) was considered the case where a visually-diagnosed healthy ibex was subsequently found to have *S. scabiei* mites either in the skin scrapings or the skin digestions. True positives (TP) or true negatives (TN) were animals properly diagnosed as infected or uninfected individuals. The explanatory variables where the same in both cases: sex (males, *n* = 210; females, *n* = 116), age class (kids, *n* = 8; yearlings, *n* = 32; juveniles, *n* = 132; adults, *n* = 148) and the period of the year [dry (summer and autumn), *n* = 99; wet (winter and spring), *n* = 246]. Model selection was based on the lowest Akaike information criterion (AIC) [36].

Finally, SP and SE estimations were based on Altman & Bland [37]:

Sensitivity = TP / TP + FN

Specificity = TN / TN + FP

Statistical procedures were performed using the R software 3.2.2 version.

## Results

SE and SP of the visual determination of mangy animals were 87.14 and 60.71%, respectively. The model selection indicated age class and period of the year as the main factors explaining SE (Table 1), while sex and period of the year were main factors for SP (Table 2).

**Table 1:** Model selection for exploring the best generalized linear model (GLM) explaining sensitivity variance of the visual detection of mange in 347 Iberian ibexes. In bold, the model with substantial support for being the best model

GLM	K	AIC	ΔAIC	Wi
**AgeClass + Period**	**5**	**182.25**	**0.00**	**0.58**
Sex × Period + AgeClass	7	183.86	1.75	0.24
Sex × AgeClass + Period	9	184.99	3.05	0.13
AgeClass × Period	8	187.91	5.88	0.03
Sex + Period	3	190.51	8.17	< 0.01
Sex × Period	4	190.62	8.33	< 0.01
Period	2	194.44	12.07	< 0.01
AgeClass	4	196.95	14.65	< 0.01
Sex + AgeClass	5	198.65	16.40	< 0.01
Sex × AgeClass	8	200.71	18.68	< 0.01
Sex	2	203.46	21.09	< 0.01

GLM: Generalized linear model; K: degrees of freedom, estimated parameters in the model; AIC: Akaike’s information criterion; ΔAIC: difference of AIC with respect to the best model; Wi: Akaike’s weight

**Table 2:** Model selection for exploring the best generalized linear model (GLM) explaining specificity variance of the visual detection of mange in 347 Iberian ibexes. In bold, the model with substantial support for being the best model

GLM	K	AIC	ΔAIC	Wi
**Sex + Period**	**3**	**209.91**	**0.00**	**0.42**
Sex × Period	4	211.24	1.37	0.21
Period	2	211.57	1.63	0.19
Sex	2	213.33	3.39	0.08
AgeClass + Period	5	214.27	4.45	0.04
Sex × Period + AgeClass	7	215.11	5.43	0.03
AgeClass × Period	8	217.21	7.60	< 0.01
Sex + AgeClass	5	217.57	7.75	< 0.01
Sex × AgeClass + Period	9	218.76	8.89	< 0.01
AgeClass	4	218.76	8.89	< 0.01
Sex × AgeClass	8	222.61	13.00	< 0.01

GLM: Generalized linear model; K: degrees of freedom, estimated parameters in the model; AIC: Akaike’s information criterion; ΔAIC: difference of AIC with respect to the best model; Wi: Akaike’s weight

The best model for SE included the additive effects of age class and period of the year, explaining 13.84% of the observed variance (Table 1). In particular, juveniles were more prone to be false negative than adults (*β*_juveniles_ = 1.4603 ± 0.4716, *Z* = 3.096, *P* = 0.00196) and ibexes were more likely to be detected as false negatives during the dry period than the wet period (*β*_wet_ _period_ = − 1.6736 ± 0.4104, *Z* = − 4.078, *P* = 4.55e^−05^). Specifically, juveniles [odds ratio (OR) = e^1.46^ = 4.30] were 4.30 times more likely to be false negative than adults, and in the dry period (OR = 1/0.19 = 5.26) the probability of false negative diagnosis was 5.26 higher than in the wet period (OR = e^−1.67^ = 0.19). Hence, SE was significantly higher in the wet period than in the dry one (92.01 *vs* 73.44%, *P* < 0.001) and for adults as compared to juveniles (94.26 *vs* 64.76%, *P* < 0.01) (Table 3). No significant differences in SE were observed for kids and yearlings.

**Table 3:** Sensitivity and specificity of the visual diagnosis of sarcoptic mange in 347 Iberian ibexes (kid, yearling, juvenile and adult) culled in Sierra Nevada Natural Space during the wet (winter and spring) and the dry periods (summer and autumn) of the year. *P*-values were obtained from the generalized linear models (GLMs) proposed to explain the sensitivity and specificity, which included the variables age, sex and period of the year

	Parameters involved in the GLMs		*P*-value
Sensitivity (%)	Dry period (73.44)	Wet period (92.09)	< 0.0001
	Juveniles (64.76)	Adults (94.26)	0.00196
Specificity (%)	Wet period (55.22)	Dry period (82.35)	0.0443
	Females (22.73)	Males (74.19)	0.0643

The best model for SP included the additive effects of sex and period of the year, explaining 4.39% of the variance (Table 2). Specifically, females were more often detected as false positives than males (*β*_male_ =-0.6896 ± 0.3727, *Z* = -1.850, *P* = 0.0643) and in the wet period individuals were more likely detected as false positives than in the dry period (*β*_wet period_ =1.2503 ± 0.6218, *Z* = 2.011, *P* = 0.0443).

Females (OR = 1/0.50 = 2) are two times more probable to be found as a false positive than males (OR = e^−0.69^= 0.50) and ibexes spotted in the wet period are 3.49 times more likely to be diagnosed as false positive than in the dry period (OR = e^1.25^ = 3.49). Thus, visual diagnosis SP was significantly higher for males than females (74.19 *vs* 22.73%, *P* < 0.1) and in the dry period compared to the wet period (82.35 *vs* 55.22%, *P* < 0.05; Table 3).

## Discussion

Visual diagnosis of sarcoptic mange aimed at managing the disease through selective culling is more sensitive than specific, resulting in a neglected bias seeking for sick ibexes in field conditions. These results contrast with previous studies reporting a SE of 60% and a SP of 100% for visual diagnosis of sarcoptic mange in the same host species [31]. However, the fact that no skin digestions in healthy individuals were performed by Arenas et al. [31] hampers any comparison with our results. Moreover, in diagnosis based on clinical signs and lesions, SE depends on the threshold established to consider a positive diagnosis and SP has been observed to be low in human scabies studies. Generally, even by adding skin scrapings to visual diagnosis, SE below 50% is common in the scientific literature [38, 39].

High relative humidity and low temperatures favour *S. scabiei* survival [17, 40], especially when they are off the host, directly affecting the viability of the mite [18, 41]. In experimental infestations, lesions were more pronounced in moistened areas of skin, increasing the weakness of the stratum corneum, facilitating mite burrowing [42]. Thus, in a wet and colder environment more severe lesions can be seen than in dry conditions with the same number of mites [25], and mites commonly increase in number as they are favoured by the weather. Conversely, summer is the most unfavourable season for *S. scabiei* [17], as high temperatures and low humidity may cause their early death [18]. Accordingly, sarcoptic mange usually reaches its highest prevalence in winter and spring (the wet period) [17], not only in the Iberian ibex but also in other species, such as the red deer (*Cervus elaphus*) and Iberian wolf (*Canis lupus signatus*) [40, 43]. These environmental effects on mites are normally reflected in the clinical status of mange about 2–3 months afterwards [17]. The higher SE and lower SP during the wet period, when mites are more active and lesions are typically more evident, suggest that such wide lesions might be more easily detected, but also that other skin disorders can be more easily mistaken as sarcoptic mange at that time of the year. In fact, in late spring, as observed in other mountain ungulates [44], Iberian ibexes are molting and can show an scabietic aspect increasing the likelihood of being wrongly culled for that condition. Along the same lines, skin disturbances due to other ectoparasite infestations can also alter the skin appearance at that time [45, 46], increasing the probability of misclassification. Therefore, during the wet period attention should be paid to distinguish mangy ibexes from those suffering from other skin disorders. Conversely, during the dry period the efforts should focus on identifying smaller lesions in otherwise apparently healthy ibexes.

In sexually dimorphic species, males are more susceptible to most parasite infestations [47, 48]. Moreover, sex-biased parasitism by arthropods rises with the increase of sexual dimorphism in body mass [48]. Although high testosterone and high investment in secondary sexual characters increase mating success, they also impair the functioning of the immune system [21], specifically under environmental stress [49]. Thus, males often show higher prevalence, parasite intensity [31, 47–50] and mortality [49], and females are normally the immunological stronger gender [21]. Although some species, such as wolves or chamois, do not show sex-bias for the presence of mange-compatible lesions [11, 24, 43], in other species such as the red deer or aoudad (*Ammotragus lervia*) sarcoptic mange is more prevalent in males than in females [16, 40]. Similarly, sex is a determining factor for the immune response to sarcoptic mange in Iberian ibexes. Females have higher specific acquired response to infestation and re-infestation than males [22]. In addition, female Iberian ibexes show lower mange prevalence, better body condition when infested and are less prone to develop severe stages of mange than males [20]. The lower prevalence and milder lesions of females may explain the lower SP of visual diagnosis of sarcopic mange, increasing the difficulty to identify them as scabietic. Conversely, more prevalent and more often severely affected males are more likely to be correctly classified as affected by sarcoptic mange, showing a better SP.

Lower prevalence of mange-compatible lesions in juveniles as compared to adults has been repeatedly reported in several species, including Iberian wolves [38] and aoudad [16]. Juvenile Iberian ibexes also appear to show lower morbidity [12] and progression to chronic stages of infection [13], although no differences in immune response to sarcoptic mange have been observed for this age class [22]. In mortality-based studies, the lower proportion of scabietic juveniles has been explained by the lower detectability of the smaller juvenile carcasses and the action of scavengers [11, 40]. The higher proportion of severe mangy adults, however, could be explained by the behaviour of the adults during the rutting season, which increases the contact among them favouring mange transmission. Since the results of the present study show that the sensitivity of visual diagnosis of sarcoptic mange in juveniles is lower than in adults, the lower prevalence consistently observed in juveniles could be also related, at least partially, to the higher rate of false negatives in this age group.

Culling animals affected by sarcoptic mange in order to decrease transmission and control the disease is a management measure that must be applied carefully and responsibly in order to achieve the objective. Otherwise, selective culling might be disadvantageous and even counter-productive due to the removal of potential resilient animals from the environment [7], disrupting the transmission of the genetic resistance to the offspring and increasing sarcoptic mange cases by enhancing animal dispersion [7, 22].

## Conclusions

The SE and SP for the visual determination of mangy Iberian ibexes were found to be influenced by physiological (sex and age) and environmental conditions in SNNS. If the selective culling of mangy individuals is to be an effective and efficient management measure, it is essential to maximize the SE and SP in order to achieve a high rate of removal of affected individuals from the environment without eliminating potentially resistant individuals. To improve the SE and SP, adaptive criteria must be established and modified according to the period of the year (wet or dry), the sex and the age of the target ibex. Culling should be more conservative during the wet period and with females, when visual diagnosis SP is low. Females seem to be more able to control sarcoptic mange, maintaining lower mite burdens and being more sedentary, therefore downplaying their role in the transmission. On the other hand, juveniles are frequently asymptomatic carriers and thus the decision of keeping alive young ibexes showing sarcoptic mange compatible lesions but potentially resistant to the disease need to be discussed. Research to unveil the reason for the lower lesions presence in juveniles and to define new field methods with higher SE for juveniles should be carried out. Similarly, new methods should be validated to unmask the false negatives during the dry period, before the rutting season when sarcoptic mange spreads due to the increase in contact rate among individuals.

## Data Availability

The datasets used and/or analysed during the present study are available upon request.

## Abbreviations

KOH: potassium hydroxide
SP: specificity
SE: sensitivity
SNNS: Sierra Nevada Natural Space
GLM: generalized linear model
K: estimated parameters in the model, degrees of freedom
AIC: Akaike’s information criterion
ΔAIC: difference of AIC with respect to the best model
Wi: Akaike’s weight
AICc: Akaike’s information criterion corrected
OR: odds ratio
TP: true positives
FN: false negatives
TN: true negatives
FP: false positives

## References

[1] Bornstein S, Mörner T, Samuel WM, Samuel WM, Pybus MJ, Kocan AA (2001). *Sarcoptes scabiei* and sarcoptic mange. Parasitic diseases of wild mammals.

[2] Pence DB, Ueckermann E (2002). Sarcoptic mange in wildlife. Rev Sci Tech.

[3] Ryser-Degiorgis MP, Ryser A, Bacciarini LN, Angst C, Gottstein B, Janovsky M (2002). Notoedric and sarcoptic mange in free-ranging lynx from Switzerland. J Wildl Dis.

[4] Pence DB, Windberg LA (1994). Impact of a sarcoptic mange epizootic on a coyote population. J Wildl Manage..

[5] Gakuya F, Ombui J, Maingi N, Muchemi G, Ogara W, Soriguer RC (2012). Sarcoptic mange and cheetah conservation in Masai Mara (Kenya): epidemiological study in a wildlife/livestock system. Parasitology.

[6] Mörner T (1992). Sarcoptic mange in Swedish wildlife. Rev Sci Tech.

[7] Lindstrom E, Mörner T (1985). The spreading of sarcoptic mange among Swedish red foxes (*Vulpes vulpes* L) in relation to fox population dynamics. Ecology.

[8] Gray DF (1937). Sarcoptic mange affecting wild fauna in New South Wales. Aust Vet J.

[9] Martin RW, Handasyde KA, Skerratt LF (1998). Current distribution of sarcoptic mange in wombats. Aust Vet J.

[10] Brown AS, Seawwright AA, Wilkinson GT (1982). The use of amitraz in the control of an outbreak of sarcoptic mange in a colony of koalas. Aust Vet J.

[11] Fernández-Morán J, Gómez S, Ballesteros F, Quirós P, Benito JL, Feliu C (1997). Epizootiology of sarcoptic mange in a population of cantabrian chamois (*Rupicapra pyrenaica parva*) in northwestern Spain. Vet Parasitol.

[12] Fandos P (1991). La cabra montés (*Capra pyrenaica*) en el Parque Natural de las Sierras de Cazorla, Segura y las Villas.

[13] León-Vizcaíno L, Ruíz de Ybáñez MR, Cubero MJ, Ortíz JM, Espinosa J, Pérez L (1999). Sarcoptic mange in Spanish ibex from Spain. J Wildl Dis.

[14] Alasaad S, Granados JE, Fandos P, Cano-Manuel FJ, Soriguer RC, Pérez JM (2013). The use of radio-collars for monitoring wildlife diseases: a case study from Iberian ibex affected by *Sarcoptes scabiei* in Sierra Nevada, Spain. Parasit Vectors.

[15] Espinosa J, Ráez-Bravo A, López-Olvera JR, Pérez JM, Lavín S, Tvarijonaviciute A (2017). Histopathology, microbiology and the inflammatory process associated with *Sarcoptes scabiei* infection in the Iberian ibex, *Capra pyrenaica*. Parasit Vectors.

[16] González-Candela M, Léon-Vizcaíno L, Cubero-Pablo MJ (2004). Population effects of sarcoptic mange in Barbary sheep (*Ammotragus Lervia*) from Sierra Espuña Regional Park. Spain. J Wildl Dis.

[17] Pérez JM, Ruiz-Martinez I, Granados JE, Soriguer RC, Fandos P (1997). The dynamics of sarcoptic mange in the ibex population of Sierra Nevada in Spain - Influence of climatic factors. J Wildl Res.

[18] Arlian LG, Runyan RA, Achar S, Estes SA (1984). Survival and infestivity of *Sarcoptes scabiei* var. *canis* and var. *hominis*. J Am Acad Dermatol.

[19] Arlian LG, Runyan RA, Vyszenski-Moher DL (1988). Water balance and nutrient procurement of S*arcoptes scabiei* var. *canis* (Acari: Sarcoptidae). J Med Entomol.

[20] López-Olvera JR, Serrano E, Armenteros A, Pérez JM, Fandos P, Carvalho J (2015). Sex-biased severity of sarcoptic mange at the same biological cost in a sexually dimorphic ungulate. Parasit Vectors.

[21] Folstad I, Karter AJ (1992). Parasites, bright males, and the immunocompetence handicap. Am Nat.

[22] Sarasa M, Rambozzi L, Rossi L, Meneguz PG, Serrano E, Granados JE (2010). *Sarcoptes scabiei*: specific immune response to sarcoptic mange in the Iberian ibex *Capra pyrenaica* depends on previous exposure and sex. Exp Parasitol.

[23] Granados JE, Montes J, Pérez JM, Serrano E, Fandos P, Soriguer RC. Plan de gestión de la cabra montés (*Capra pyrenaica*) en el Espacio Natural Protegido de Sierra Nevada. 2001.

[24] Rossi L, Fraquelli C, Vesco U, Permunian R, Sommavilla GM, Carmignola G (2007). Descriptive epidemiology of a scabies epidemic in chamois in the Dolomite Alps, Italy. Eur J Wildl Res.

[25] Pérez JM, Granados JE, Sarasa M, Serrano E (2011). Usefulness of estimated surface area of damaged skin as a proxy of mite load in the monitoring of sarcoptic mange in free-ranging populations of Iberian wild goat, *Capra pyrenaica*. Vet Parasitol.

[26] Pérez JM, Castro I, Granados JE, Cano-Manuel FJ, Fandos P, Espinosa J (2016). Does *Sarcoptes scabiei* synchronize its breeding cycle with that of the Iberian Ibex, *Capra pyrenaica*?. Int J Acarol.

[27] Pérez JM, Granados J, Gomez F, Pérez C, Ruiz-Martínez I (1996). Las parasitosis de la cabra montes de Sierra Nevada (Granada). Conf Int Sierra Nevada Conserv y Desarro Sosten.

[28] Pérez JM, Meneguz PG, Dematteis A, Rossi L, Serrano E (2006). Parasites and conservation biology: The “ibex-ecosystem”. Biodivers Conserv.

[29] Smith MC, Sherman DM (2009). Goat medicine.

[30] Heukelbach J, Feldmeier H (2006). Scabies. Lancet.

[31] Arenas AJ, Gómez F, Salas R, Carrasco P, Borge C, Maldonado A (2002). An evaluation of the application of infrared thermal imaging to the tele-diagnosis of sarcoptic mange in the Spanish ibex (*Capra pyrenaica*). Vet Parasitol..

[32] Carvalho J, Granados JE, López-Olvera JR, Cano-Manuel FJ, Pérez JM, Fandos P (2015). Sarcoptic mange breaks up bottom-up regulation of body condition in a large herbivore population. Parasit Vectors.

[33] Pérez JM (2001). Distribución, genética y estatus sanitario de las poblaciones andaluzas de cabra montés.

[34] Serrano E, Granados JE, Sarasa M, González FJ, Fandos P, Soriguer RC (2011). The effects of winter severity and population density on body stores in the Iberian wild goat (*Capra pyrenaica*) in a highly seasonal mountain environment. Eur J Wildl Res.

[35] Ráez-Bravo A, Granados JE, Cerón JJ, Cano-Manuel FJ, Fandos P, Pérez JM (2015). Acute phase proteins increase with sarcoptic mange status and severity in Iberian ibex (*Capra pyrenaica* Schinz, 1838). Parasitol Res.

[36] Burnham KP, Anderson DR (2002). Model selection and multimodel inference. A practical information-theoretic approach.

[37] Altman DG, Bland JM (1994). Diagnostic tests 1: sensitivity and specificity. Br Med J..

[38] Walter B, Heukelbach J, Fengler G, Worth C, Hengge U, Feldmeier H (2011). Comparison of dermoscopy, skin scraping, and the adhesive tape test for the diagnosis of scabies in a resource-poor setting. Arch Dermatol.

[39] Walton SF, Currie BJ (2007). Problems in diagnosing scabies, a global disease in human and animal populations. Clin Microbiol Rev.

[40] Oleaga Á, Casais R, González-Quirós P, Prieto M, Gortázar C (2008). Sarcoptic mange in red deer from Spain: improved surveillance or disease emergence?. Vet Parasitol.

[41] Arlian LG, Pole MJ, Vyszensky-Moher DL (1989). Survival of adults and developmental stages of *Sarcoptes scabiei* var. *canis* when off the host. Exp Appl Acarol.

[42] Ibrahim KEE, Abu-Samra MT (1987). Experimental transmission of a goat strain of *Sarcoptes scabiei* to desert sheep and its treatment with ivermectin. Vet Parasitol..

[43] Oleaga Á, Casais R, Balseiro A, Espí A, Llaneza L, Hartasánchez A (2011). New techniques for an old disease: sarcoptic mange in the Iberian wolf. Vet Parasitol.

[44] Déry F, Hamle S, Côté S (2019). Gretting ready for the winter: timing and determinants of molt in alpine ungulate. Ecol Evol.

[45] Varela-Castro L, Zuddas C, Ortega N, Serrano E, Salinas J, Castellà J (2018). On the possible role of ticks in the eco-epidemiology of *Coxiella burnetii* in a Mediterranean ecosystem. Tick Tick Borne Dis.

[46] Carvalho J, Serrano E, Pettorelli N, Granados JE, Habela MA, Olmeda S (2016). *Sarcoptes scabiei* infestation does not alter the stability of ectoparasite communities. Parasit Vectors.

[47] Klein SL (2004). Hormonal and immunological mechanisms mediating sex differences in parasite infection. Parasite Immunol..

[48] Moore SL, Wilson K (2002). Parasites as a viability cost of sexual selection in natural populations of mammal. Science.

[49] Toïgo C, Gaillard JM (2003). Causes of sex biased adult survival in ungulates: sexual size dimorphism, mating tactic or environment harshness?. Oikos.

[50] Decristophoris PMA, Von Hardenberg A, McElligott AG (2007). Testosterone is positively related to the output of nematode eggs in male Alpine ibex (*Capra ibex*) faeces. Evol Ecol Res.

